# Antimicrobial activity of a natural compound and analogs against multi-drug-resistant Gram-positive pathogens

**DOI:** 10.1128/spectrum.01515-22

**Published:** 2024-01-30

**Authors:** Kush N. Shah, Parth N. Shah, Francesca O. Agobe, Kaitlyn Lovato, Hongyin Gao, Oluwadara Ogun, Cason Hoffman, Marium Yabe-Gill, Qingquan Chen, Jordan Sweatt, Bhagath Chirra, Ricardo Muñoz-Medina, Delaney E. Farmer, László Kürti, Carolyn L. Cannon

**Affiliations:** 1 Department of Microbial Pathogenesis & Immunology, College of Medicine, Texas A&M University Health Science Center, Bryan, Texas, USA; 2 Department of Chemistry, Rice University, Houston, Texas, USA; 3 Department of Pediatrics, University of Texas Southwestern Medical Center, Dallas, Texas, USA; University of Nebraska Medical Center, Omaha, Nebraska, USA

**Keywords:** natural product, MRSA, antibiotic

## Abstract

The increasing prevalence of methicillin-resistant *Staphylococcus aureus* (MRSA) has sparked global concern due to the dwindling availability of effective antibiotics. To increase our treatment options, researchers have investigated naturally occurring antimicrobial compounds and have identified MC21-A (C58), which has potent antimicrobial activity against MRSA. Recently, we have devised total synthesis schemes for C58 and its chloro-analog, C59. Here, we report that both compounds eradicate 90% of the 39 MRSA isolates tested [MIC_90_ and minimum bactericidal concentration (MBC_90_)] at lower or comparable concentrations compared to several standard-of-care (SoC) antimicrobials including daptomycin, vancomycin, and linezolid. Furthermore, a stable, water-soluble sodium salt of C59, C59Na, demonstrates antimicrobial activity comparable to C59. C59, unlike vancomycin, kills stationary-phase MRSA in a dose-dependent manner and completely eradicates MRSA biofilms. In contrast to vancomycin, exposing MRSA to sub-MIC concentrations of C59 does not result in the emergence of spontaneous resistance. Similarly, in a multi-step study, C59 demonstrates a low propensity of resistance acquisition when compared to SoC antimicrobials, such as linezolid and clindamycin. Our findings suggest C58, C59, and C59Na are non-toxic to mammalian cells at concentrations that exert antimicrobial activity; the lethal dose at median cell viability (LD_50_) is at least fivefold higher than the MBC_90_ in the two mammalian cell lines tested. A morphological examination of the effects of C59 on a MRSA isolate suggests the inhibition of the cell division process as a mechanism of action. Our results demonstrate the potential of this naturally occurring compound and its analogs as non-toxic next-generation antimicrobials to combat MRSA infections

**IMPORTANCE:**

The rapid emergence of methicillin-resistant *Staphylococcus aureus* (MRSA) isolates has precipitated a critical need for novel antibiotics. We have developed a one-pot synthesis method for naturally occurring compounds such as MC21-A (C58) and its chloro-analog, C59. Our findings demonstrate that these compounds kill MRSA isolates at lower or comparable concentrations to standard-of-care (SoC) antimicrobials. C59 eradicates MRSA cells in biofilms, which are notoriously difficult to treat with SoC antibiotics. Additionally, the lack of resistance development observed with C59 treatment is a significant advantage when compared to currently available antibiotics. Furthermore, these compounds are non-toxic to mammalian cell lines at effective concentrations. Our findings indicate the potential of these compounds to treat MRSA infections and underscore the importance of exploring natural products for novel antibiotics. Further investigation will be essential to fully realize the therapeutic potential of these next-generation antimicrobials to address the critical issue of antimicrobial resistance.

## INTRODUCTION

In the past two decades, the convergence of a shrinking antibiotic pipeline and the rapid emergence of antimicrobial resistance have led to a severe shortage of strategies to combat resistant bacterial infections. In the United States, the Centers for Disease Control and Prevention estimates that more than 2.8 million cases of serious antibiotic-resistant infections occur annually, resulting in at least 35,000 fatalities (1, 2). Methicillin-resistant *Staphylococcus aureus* (MRSA) is the cause of 323,700 serious infections and 10,600 deaths (1, 2). MRSA, known for its ability to infect soft tissues and form biofilms on implants, is resistant to many antibiotics currently available (3–6). The increasing prevalence of MRSA isolates indicates that there is a critical need for novel antimicrobials.

To address the formidable challenges associated with the treatment of MRSA infections, extensive modifications have been made to existing antibiotics to maintain their antimicrobial activity (7). However, these modifications are often associated with limitations, and benefits are short-lived, as evidenced by the emergence of isolates resistant to the current standard of care (SoC), vancomycin, which include vancomycin-intermediate *S. aureus* and vancomycin-resistant *S. aureus* (8, 9). Despite alternatives to vancomycin including some recently developed antibiotics (e.g., ceftaroline, clindamycin, dalbavancin, daptomycin, delafloxacin, doxycycline, linezolid, minocycline, omadacycline, oritavancin, tedizolid, teicoplanin, telavancin, tigecycline, and trimethoprim/sulfamethoxazole), treating MRSA infections remains a challenge. The ability of MRSA to form biofilms coupled with its propensity to develop resistance has rendered many currently available antibiotics obsolete. Thus, it is critical to develop small-molecule therapeutics that target unexplored mechanisms to eradicate this pathogen. Recently, several reports have identified naturally occurring compounds, isolated from sources such as fruit extracts and other bacteria, that demonstrate antimicrobial activity against a wide spectrum of bacterial pathogens (9–15). One such compound, MC21-A (C58), isolated from a marine bacterium, *Pseudoalteromonas phenolica*, demonstrates potent antimicrobial activity against clinical MRSA strains. However, cumbersome biosynthesis and poor yields have hampered the exploration of this compound as a viable, next-generation antimicrobial agent (12). We have devised a total synthesis scheme, which has revolutionized our ability to synthesize highly targeted compounds including naturally occurring molecules, including C58 (16). Specifically, with the use of bromine or thionyl chloride as halogenating agents, complete halogenation at both the *ortho* and *para* positions of 2,2′-biphenol can be achieved, without any observable selective ortho halogenation by-products. This technique readily allows for scaling the synthesis of this compound. Here, we have synthesized C58 and a chloro-analog of C58, C59, using this economical and operationally simple synthesis technique. Next, we evaluated the antimicrobial activity of both compounds and their water-soluble salts against a panel of clinical and laboratory MRSA isolates in planktonic and biofilm growth modes. We have determined the propensity of resistance acquisition using single-step and multi-step *in vitro* assays. We have defined the selectivity index through the evaluation of the toxicity of these compounds against human bronchial epithelial (16HBE) and human dermal fibroblast (HDF) cell lines. Finally, we have performed a preliminary investigation of the mechanism of action of these novel antimicrobials.

## RESULTS

### pH and *K*_a_ data for the investigated compounds

The pH of a 10-g/L solution of C58, C59, the salts of C59 (i.e., C59Na, C59Ca, C59Li, and C59K), and C59Ac in distilled, deionized water was determined. The acid dissociation constants (*K*_a_) for the solutions of C58 and C59 were 6.80 (*K*_a_ = 1.26 × 10^−12^) and 9.86 (*K*_a_ = 6.18 × 10^−19^), respectively. Solutions of the lithium and potassium salts (C59Li and C59K) had pH values greater than 10 and smaller *K*_a_ constants than C59 and the other salts. These findings indicated that C59Li and C59K would exhibit less dissociation than the other compounds, which would likely lead to the lower availability of the phenol (O^−^) anion, a factor we believe is important for antimicrobial activity. Therefore, these compounds were not included in the more detailed studies. The pH/*K*_a_ values for C59Na, C59Ac, and C59Ca were as follows: 8.69 (*K*_a_ = 1.54 × 10^−16^), 6.61 (*K*_a_ = 2.45 × 10^−12^), and 7.24 (*K*_a_ = 1.26 × 10^−13^). Although C59Ca had a promising *K*_a_ constant, this compound demonstrated minimal solubility, potentially due to the bivalent nature of the Ca^2+^ counterion, and thus, this compound was not selected for subsequent in-depth studies. C59Na and C59Ac had moderate aqueous solubility and pH and *K*_a_ constants comparable to the parent compound (C59). We anticipated that these compounds, C59Na and C59Ac, would behave similarly to C59 and hold the greatest potential for future studies.

### Antimicrobial activity

The minimum inhibitory and bactericidal concentrations (MIC and MBC) were determined for 39 clinical and laboratory isolates of MRSA. The MIC_90_ and MBC_90_ values determined are listed in Table 1. The MIC_90_ values for C58 and C59 are comparable to vancomycin, linezolid, and daptomycin and less than those of clindamycin and trimethoprim/sulfamethoxazole. The MBC_90_ values for C58 and C59 are comparable to daptomycin and less than those of all other tested antimicrobials. Furthermore, the antimicrobial activity of the derivatives of C59, water-soluble sodium (C59Na), lithium (C59Li), calcium (C59Ca), and potassium (C59K) salts, and a less polar acetate derivative, C59Ac, were evaluated against six MRSA isolates (Table S1). Among the six selected isolates, there were two isolates with MBC values greater than the respective MBC_90_ for C58, C59, and vancomycin. The acetate derivative of C59 (C59Ac) demonstrates higher MIC_90_ and MBC_90_ compared to C59, while the water-soluble sodium salts demonstrate comparable MIC_90_ concentrations and lower MBC_90_ concentrations compared to C59. To select a compound for evaluation against the full panel of 39 MRSA isolates, consideration was given to the following criteria: antimicrobial activity, the stability of the compound in solution, and the nature of the salt. Using these criteria, C59Na was selected for antimicrobial testing. The antimicrobial activity of C59Na, measured in terms of MIC_90_, was comparable to that of its parent compound, C59. C58 and C59 had potent antimicrobial activity against isolates of other Gram-positive species, but not Gram-negative species (Table S3). The Gram-negative species tested included *Enterococcus faecalis* and *Pseudomonas aeruginosa*.

**TABLE 1:** Minimum concentration resulting in the inhibition or eradication of 90% of the 39 evaluated MRSA isolates (MIC_90_ and MBC_90_) determined using the Clinical and Laboratory Standards Institute (CLSI) broth microdilution technique

Drug	MIC_90_ (µg/mL)	MBC_90_ (µg/mL)
C58	2	4
C59	2	6
C59Na	4	12
C59Ca	4	8
C59K	2	4
C59Li	4	4
Vancomycin	2	16
Linezolid	4	>32
Daptomycin	2	2
Clindamycin	>32	>32
Trimethoprim/sulfamethoxazole	24	>32

MIC and MBC values were determined against six isolates at different pH levels (Fig. S2 and S3). The pH levels were selected based on the normal physiological pH and pH levels at a site of infection, which can be as low as 5.5. With the reduction in pH, the MIC and MBC of vancomycin increased indicating less antimicrobial activity, while the MIC and MBC of C58 and C59 decreased as much as fourfold at pH 5.5 (Table S4). Like the parent compound, C59, the MIC and MBC of C59Na decreased with the reduction in pH indicating more potent antimicrobial activity, while those of C59Ac remained unchanged.

### Kill kinetics

**Fig 1:**
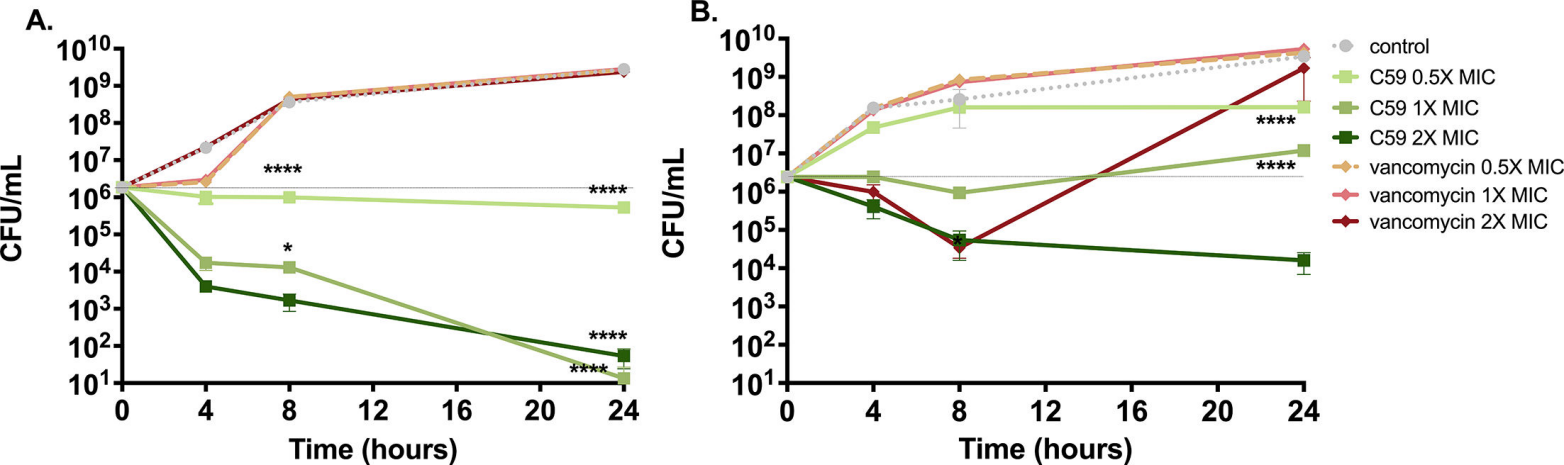
Kill kinetics of C59 or vancomycin against MRSA isolates (**A**) SA LL 06 and (**B**) TCH 1516 grown to the stationary phase. Data shown as the mean ± SD. Analyzed by two-way ANOVA with Sidak’s multiple comparison test (*n* = 10 replicates). ^**^P < 0.01; ^****^P < 0.0001. Statistical significance represented for comparisons between C59 and vancomycin at comparable concentrations for each time-point (^*^P < 0.05; ^****^P < 0.0001).

Bacterial isolates studied using standard CLSI MIC/MBC assays are incubated under static conditions. However, to assess the bactericidal activity of the compounds against isolates grown in a more dynamic environment, a kill kinetic assay was conducted. In this assay, the same quantity of bacteria was used as in the MIC/MBC CLSI standard assay; however, constant shaking and aeration accelerated bacterial growth. The rate of bacterial killing was determined against two MRSA isolates, SA LL 06 and TCH 1516 (Fig. 1). The MIC of C59 and vancomycin against SA LL 06 was 2 and 1 µg/mL, respectively, while the MIC of C59 and vancomycin against TCH 1516 was 2 and 4 µg/mL, respectively. Following incubation with stationary-phase SA LL 06, a dose response with complete bacterial eradication was observed with C59 treatment at 1× and 2× MIC concentrations after 24 hours. Conversely, 24-hour incubation with vancomycin resulted in a complete lack of bacterial inhibition. Furthermore, 8 hours of incubation with vancomycin resulted in a significantly higher bacterial burden compared to incubation with similar concentrations of C59. Analogous trends were observed with MRSA isolate TCH 1516; incubation with vancomycin for 24 hours resulted in a significantly higher bacterial burden compared with incubation with similar concentrations of C59, although incubation with C59 did not result in complete bacterial killing.

### Antimicrobial activity against bacteria in biofilm growth mode

**Fig 2:**
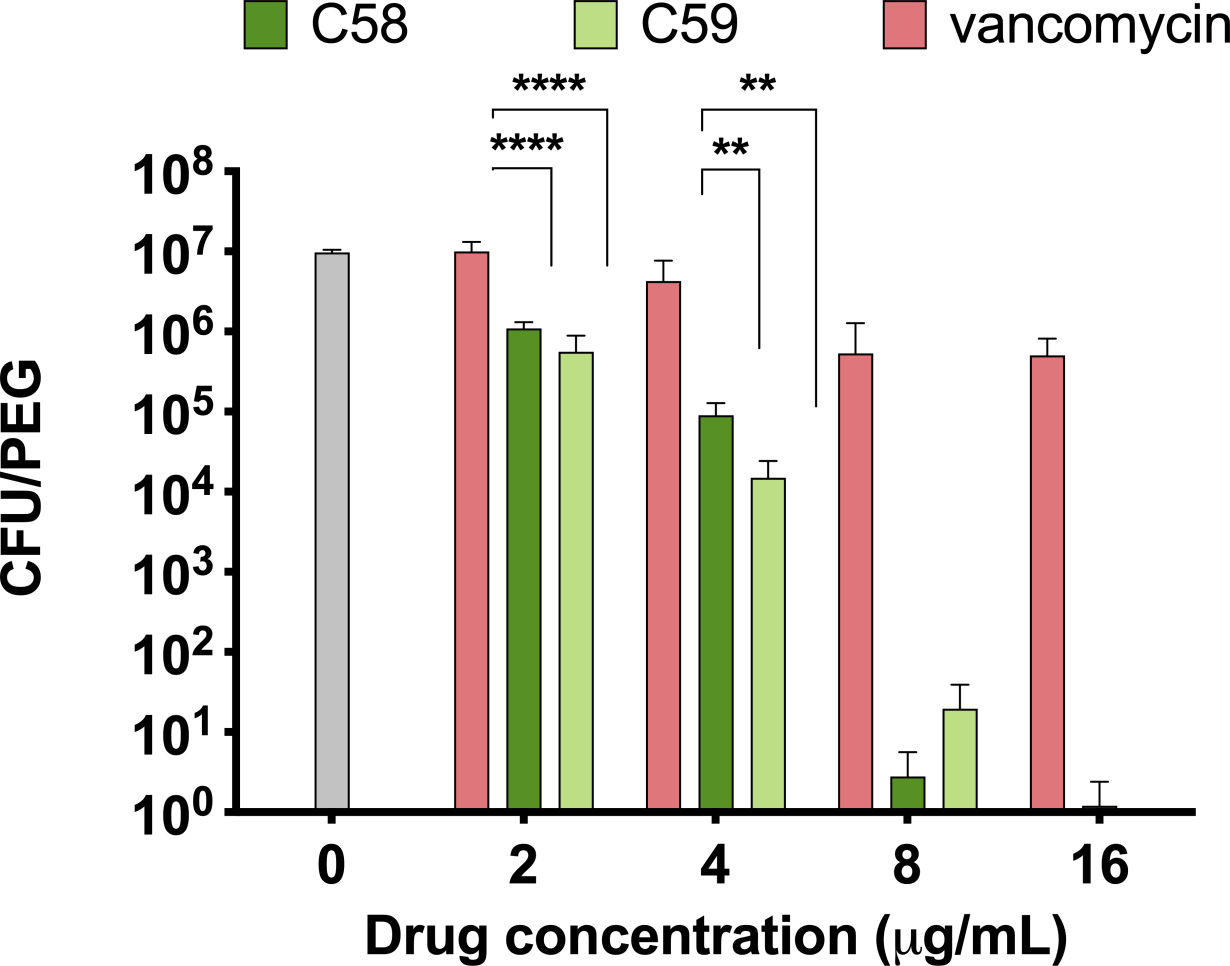
Activity of C58 and C59 compared with vancomycin against MRSA isolate, SAD05 in biofilm growth mode. Data shown as mean ± SD. Analyzed by two-way ANOVA with Sidak’s multiple comparison test (*n* = 10 replicates). ^**^P < 0.01; ^****^P < 0.0001.

Biofilm growth studies were performed to identify the MRSA isolate that formed the most robust biofilms among the 10 evaluated isolates, and SAD05 was selected for use in subsequent studies. SAD05 had a 72-hour incubation period before forming biofilms on the pegs (data not presented). The antimicrobial activity of C58 and C59 was compared with that of vancomycin against 72-hour biofilms of SAD05 (Fig. 2). Both C58 and C59 completely eradicated SAD05 biofilms at concentrations of 8 and 16 µg/mL, respectively. In contrast, vancomycin, which is still considered the gold standard for treating MRSA, did not demonstrate activity against MRSA biofilms, confirming its previously reported inefficacy in treating biofilms (17). At 2 and 4 µg/mL, there was a significant reduction in the number of bacterial colonies observed after incubating with C58 and C59 when compared with vancomycin (Fig. 2). Similar trends were observed when evaluating the antimicrobial activity of C58 and vancomycin against MRSA in the biofilm mode using a crystal violet assay. When C58 was used to treat biofilms, lower biomass was observed when compared with bacteria incubated with vancomycin (Fig. S4).

### Determination of risk of resistance acquisition

To assess the frequency of spontaneous resistance acquisition, a single-step assay was performed using C59 and vancomycin, against the MRSA isolate TCH 1516, which was grown to the stationary phase. No growth was observed when the bacterial suspension was inoculated onto plates loaded with C59 at concentrations as low as 2× MIC. In contrast, bacterial growth was observed in plates containing up to 8× MIC of vancomycin. The frequency of resistance development was determined by dividing the number of colony-forming units (CFUs) on drug-loaded plates at each concentration by the CFUs in the inoculum. The frequency of resistance acquisition upon incubation with 2×, 4×, and 8× MIC of vancomycin was 1.83 × 10^−8^ ± 7.10 × 10^−9^, 1.56 × 10^−9^ ± 7.12 × 10^−10^, and 5.77 × 10^−10^ ± 2.77 × 10^−10^, respectively.

**Fig 3:**
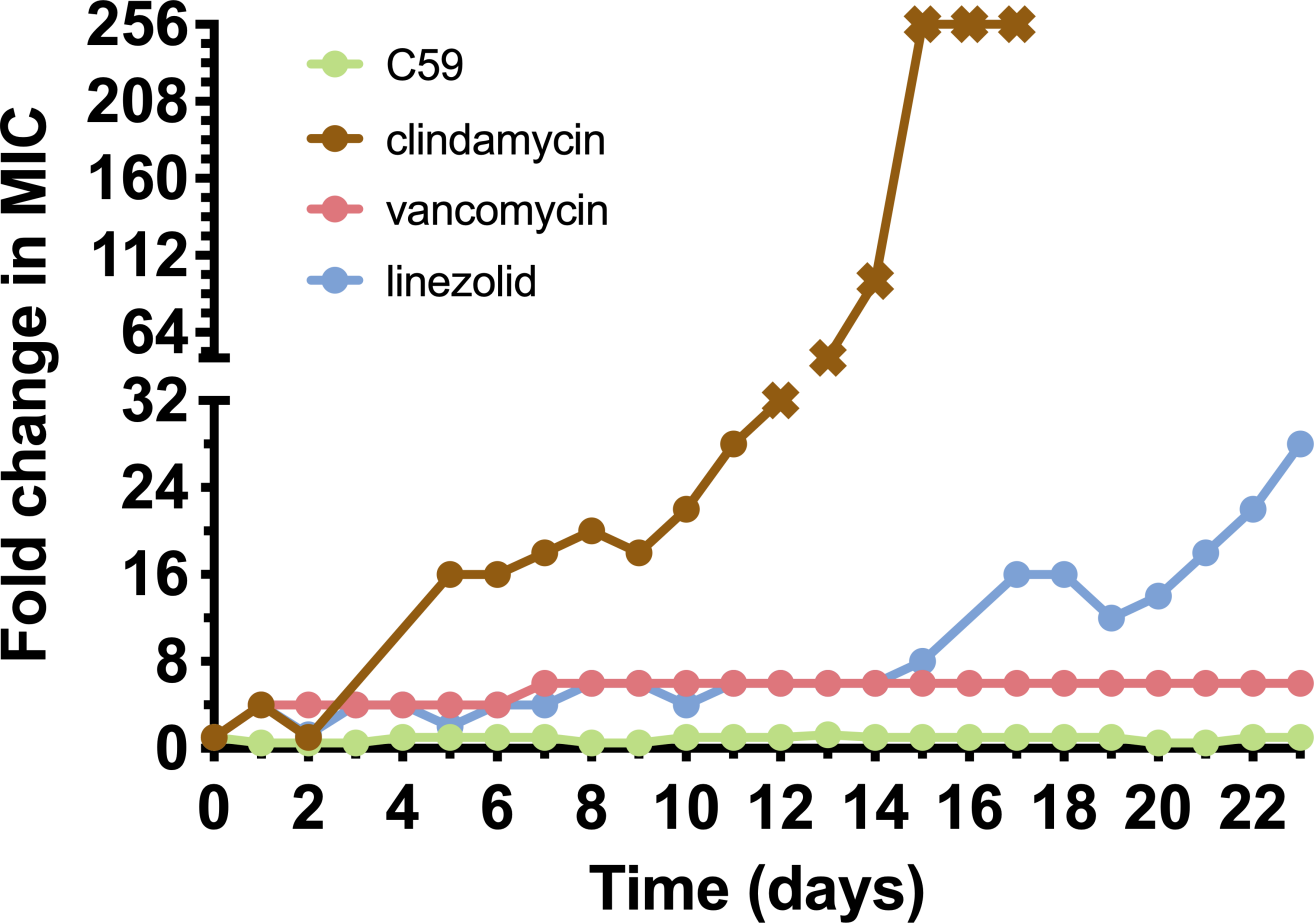
Resistance acquisition during serial passaging in the presence of sub-MIC levels of C59, clindamycin, linezolid, or vancomycin. X represents the highest concentration tested that did not result in an MIC.

A multi-step resistance acquisition study was performed to compare the change in MIC values when continuously incubated with C59 and SoC antimicrobials (clindamycin, linezolid, and vancomycin) (Fig. 3). The MIC for clindamycin increased 256-fold to 32 µg/mL after constant incubation with increasing drug concentrations after 17 days, demonstrating the ability of bacteria to rapidly acquire resistance to clindamycin. Similarly, bacteria rapidly acquired resistance to linezolid with a 16-fold increase in MIC after 21 days. Bacteria treated with vancomycin also developed resistance, with an initial increase in MIC of sixfold within 7 days. This increase was followed by stable antimicrobial activity for the remainder of the experiment, while no change in MIC was observed for bacteria treated with C59 for 21 days.

### Hemolytic activity of compounds

**Fig 4:**
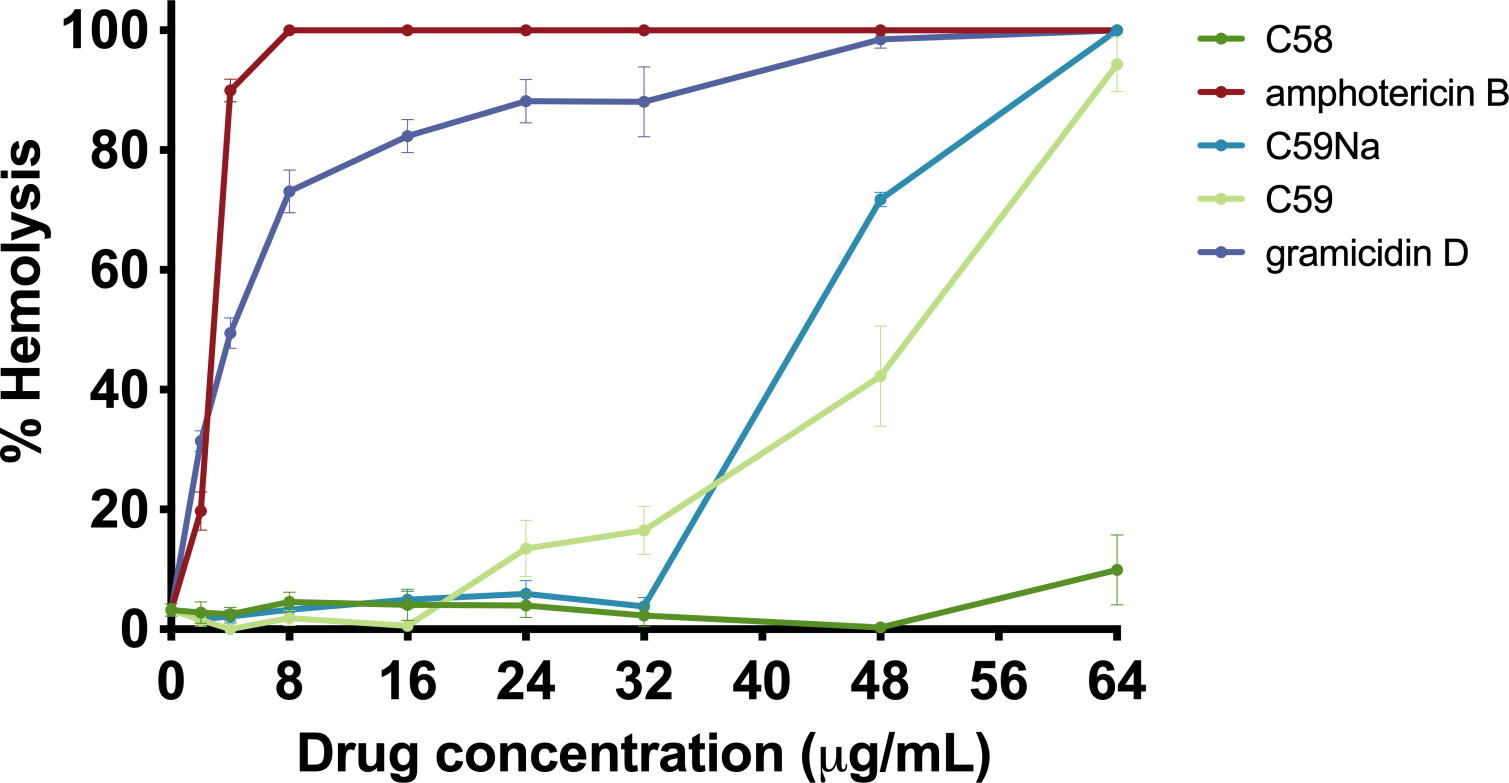
Hemolytic activity of C58, C59, and C59Na compared with amphotericin B and gramicidin D after a 1-hour incubation.

Incubation with C58 did not result in erythrocyte lysis at any of the concentrations evaluated (Fig. 4). This finding is consistent with the findings published by Isnansetyo and Kamei, the group that originally isolated C58 (12). C59 and the sodium salt of C59, C59Na, did not lyse erythrocytes at concentrations up to 24 µg/mL. Additionally, the sodium salt of C59 demonstrated similar hemolytic activity to its parent compound, C59. In contrast, amphotericin B and gramicidin D readily lysed erythrocytes at low concentrations, a trend that has been previously demonstrated (Fig. 4) (12).

### Toxicity against human bronchial epithelial and human dermal fibroblast cells

**Fig 5:**
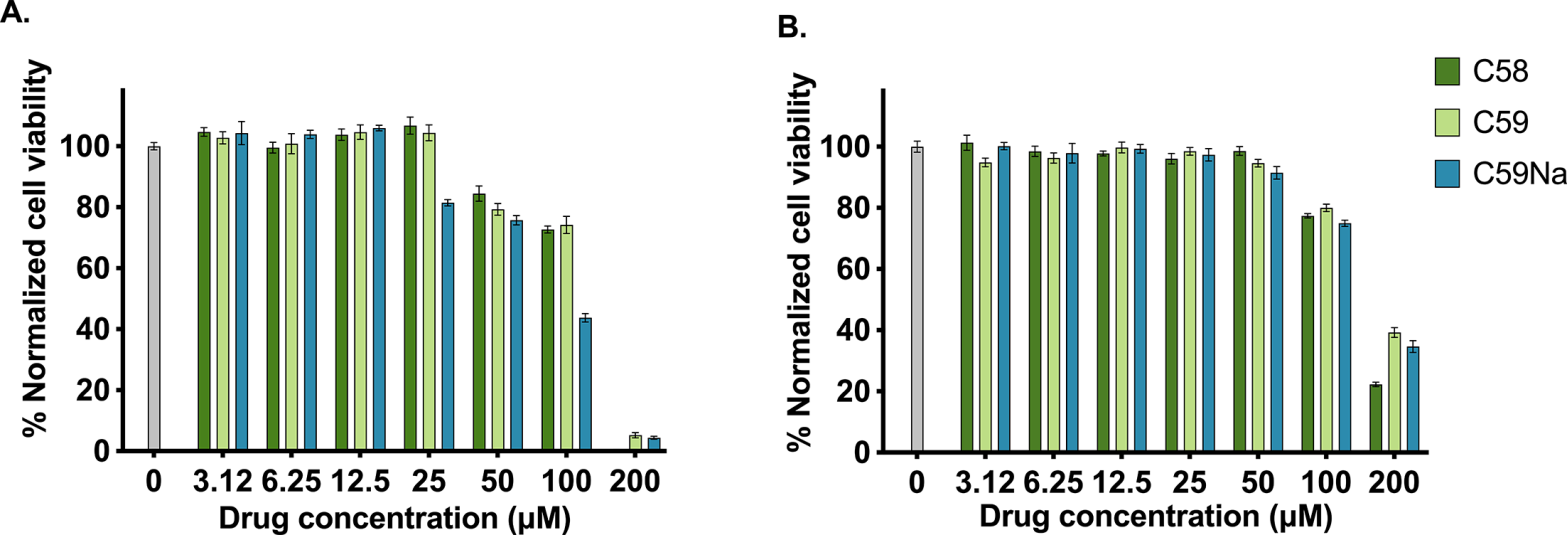
Toxicity of C58, C59, and C59Na toward (**A**) 16HBE and (**B**) HDF after a 24-hour incubation.

The toxicity of C58, C59, and C59Na against 16HBEs and HDFs with a 24-hour exposure was determined using an alamarBlue cell viability assay (Fig. 5). The LD_50_ values for all tested compounds were identified using non-linear regression analysis. These values were at least sevenfold higher than the corresponding MIC_90_ values (Table 1) for each test compound. The LD_50_ values for 16HBE cells upon 24-hour incubation with C58, C59, and C59Na were 59.3, 40.2, and 30.0 µg/mL, respectively. Similarly, the LD_50_ values for HDFs following 24-hour incubation with C58, C59, and C59Na were 71.3, 56.0, and 57.5 µg/mL, respectively. The sodium salt of C59, C59Na, demonstrated comparable toxicity to its parent compound C59 against both cell lines.

### Transmission electron microscopy

**Fig 6:**
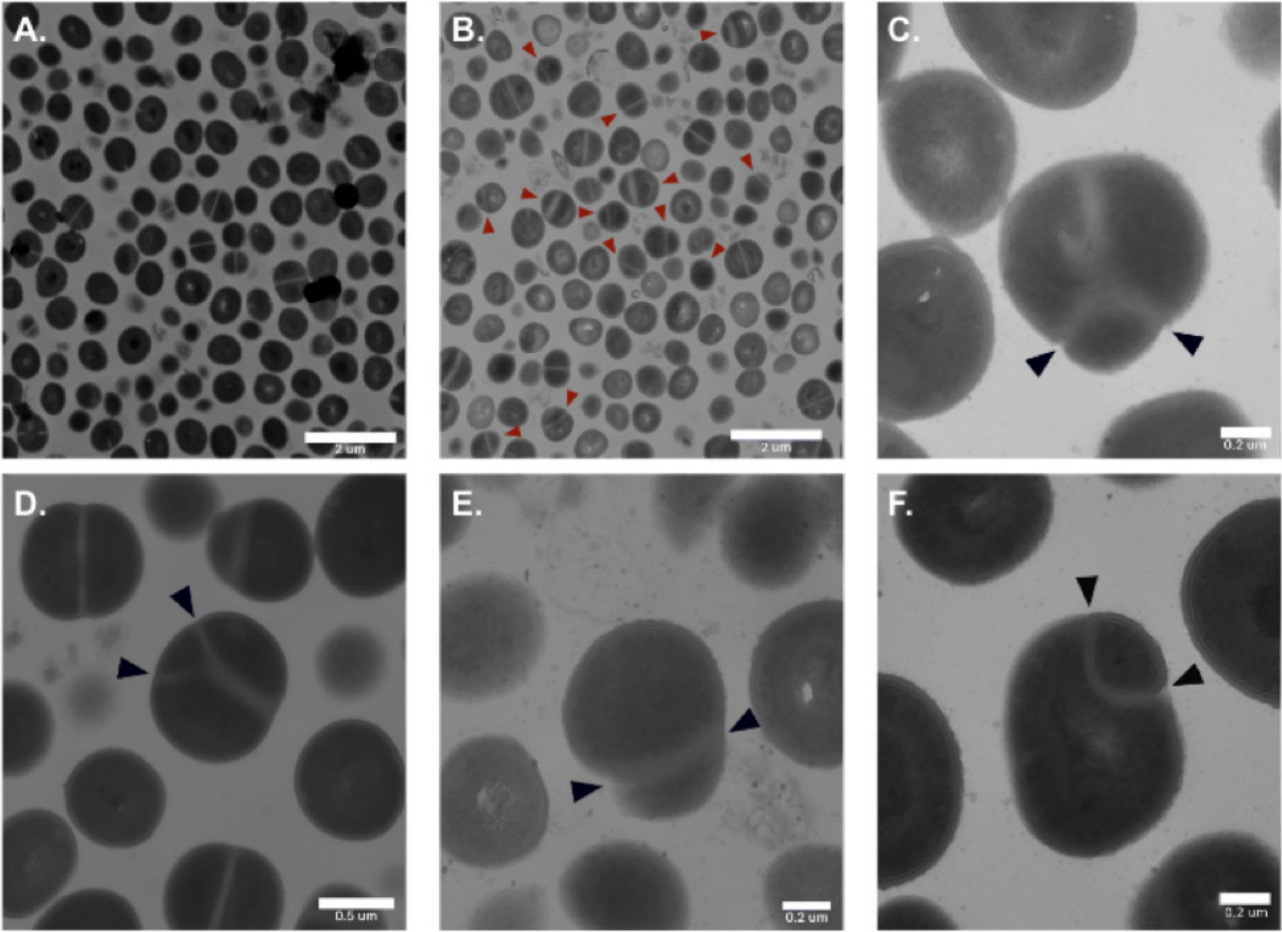
Transmission electron microscopy (TEM) images of MRSA incubated with (**A**) 0 µg/mL and (**B, C, D, E, and F**) 1 µg/mL C59. Scale bars are 2 µM for A and B, 0.5 µM for D, and 0.2 µM for C, E, and F.

MRSA isolates treated with C59 did not change in size; however, C59 treatment resulted in an increased number of diploid cells when compared with untreated bacteria (Fig. 6B). Additionally, at higher concentrations, bacteria treated with C59 demonstrated signs of membrane damage. Treatment with C59 resulted in asymmetrical cell morphology (Fig. 6B) and the formation of tetraploid bacteria with abnormal septa (Fig. 6C and D). Additionally, cells also exhibited asymmetric and irregular partitioning (Fig. 6E and F). Such morphological changes have been previously reported in live bacteria treated with antibacterial agents that target and inhibit the cell division process, such as peptide LNA787 (18).

## DISCUSSION

The rapid emergence of resistant bacterial isolates has not been met with the equally rapid development of new antibiotics, and this has resulted in a critical need for novel antimicrobials. Synthetic approaches to developing new antimicrobials have yielded limited success. As a result, researchers have shifted their focus back to the vast reservoir of naturally occurring bacterial species in the environment, which hold great promise for the discovery of novel antimicrobials (9–13). It is estimated that 99% of these bacteria have yet to be cultured in the laboratory, highlighting an area of untapped potential (10). The discovery of a species isolated from the environment, *Pseudoalteromonas phenolica,* yielded C58 (10, 12, 13). However, the isolation of bacterial species that secrete antimicrobial agents is only one piece of a multi-faceted solution. To further advance the discovery and development of new compounds and expand our antimicrobial portfolio, it is crucial to implement robust chemical synthesis and modification strategies. With this goal in mind, our collaborative initiative has devised a total synthesis scheme to produce gram quantities of C58. This scheme can be easily scaled to produce kilogram quantities of the compound. Previously reported biosynthesis techniques have proven cumbersome and could not be scaled, yielding only milligram quantities of the compound, which created a significant challenge in evaluating the compound as a potential antimicrobial therapeutic (12). Our synthetic approach inspired by the naturally occurring compound, C58, allowed us to further modify this compound and synthesize a chloro-analog (C59), as well as salts of C59, including C59Na. Here, we have characterized the compounds to determine their potential as antimicrobial therapeutics.

### Antimicrobial activity

The antimicrobial activity of C58 and C59 was evaluated against a panel of 39 MRSA isolates and compared to five SoC antimicrobials. Both compounds demonstrate comparable or more potent antimicrobial activity compared to SoC antibiotics against isolates in the planktonic growth mode. Furthermor, modification of the phenolic groups on C59 demonstrates their crucial role; blocking these phenolic groups by forming an acetate ester (C59Ac) results in reduced antimicrobial activity, while the sodium salt (C59Na) retains its antimicrobial activity. Additionally, the influence of the phenolic group on antimicrobial activity is further emphasized by the pH studies, which demonstrate that lowered pH results in increased antimicrobial activity for C58, C59, and C59Na at every pH tested. At all pH levels tested, C59 and its water-soluble formulation, C59Na, have similar antimicrobial properties. C58 also demonstrates potent antimicrobial activity at every pH tested. In contrast, the antimicrobial activity of vancomycin is reduced in acidic conditions. Thus, the tested compounds demonstrate tremendous potential as antimicrobial agents at the site of infection, where the pH is known to be as low as 5.5, due to factors including the innate immune response and an aerobic metabolism resulting in the production of lactic and acetic acids (19, 20). Furthermore, the antimicrobial activities of C58 and C59 are not limited to *S. aureus*; these compounds also demonstrated antimicrobial activity comparable to vancomycin against a panel of 21 *Staphylococcus epidermidis* isolates, as well as activity against *Enterococcus faecalis* and *Streptococcus pyogenes* (Tables S2 and S3).

### Biofilms

A key factor associated with suboptimal clinical outcomes is resistance to antimicrobial agents at clinically achievable concentrations; for example, bacteria in the biofilm growth mode often exhibit resistance to SoC antibiotics (21–24). Numerous reports have demonstrated the reduced susceptibility of bacteria in the stationary phase or biofilm growth modes to vancomycin, and this inefficacy is a significant limitation when the drug is used clinically (17, 21, 25). The reduced activity of vancomycin is accompanied by the presence of thicker cell walls in stationary-phase bacteria, which is associated with the absence of actively replicating cells (17, 26). Our results confirm previous reports indicating that vancomycin fails to exert antimicrobial activity against stationary-phase bacteria or bacteria in biofilm growth modes. In contrast, the potent antimicrobial activity of C58 and C59 is evidenced by the killing of bacteria in stationary-phase and biofilm growth modes. The results we observed with C59 mirror those published on C58 by Isnansetyo and Kamei (12). To our knowledge, the tested compounds are unique in their capacity to kill MRSA biofilms, which suggests they may have more potent activity when used to treat chronic MRSA infections than currently available antimicrobials.

### Resistance

C59 demonstrated a low probability of resistance development in assays of MRSA in the log-phase growth mode. In both single-step and multi-step resistance acquisition studies, MRSA readily developed resistance to vancomycin, but not to C59. Vancomycin demonstrated stable activity, while prolonged exposure to clindamycin or linezolid resulted in the failure of these agents to control bacterial growth. While any bacterial species may eventually develop resistance to any antibiotic over time, our findings suggest that the development of resistance to C58 and its derivatives would be delayed beyond the timeline demonstrated by SoC antibiotics. Thus, the proposed compounds address resistance associated with bacteria in log-phase and biofilm growth modes, and future studies will include the investigation of the probability of resistance development over a longer time period.

### Toxicity

In addition to confirming activity and the probability of resistance development, when characterizing an antibiotic for therapeutic applications, it is necessary to assess the safety profile of the compound. An antimicrobial agent must be non-toxic at therapeutic concentrations; thus, the potential for eukaryotic membrane damage was determined by assessing hemolytic activity against erythrocytes. Amphotericin B and gramicidin D were used as controls due to their known hemolytic activity (12). None of the tested compounds exhibit hemolytic activity at therapeutic concentrations. C58 only begins to exhibit hemolytic activity at concentrations 32-fold higher than its MIC_90_. Similarly, the LD_50_ for C59 is 24-fold higher than its MIC_90_, and the LD_50_ for C59Na is 10-fold higher than its MIC_90_. Our findings support the results reported by Isnansetyo and Kamei (12). We observed that C58 and its derivatives exhibit a safety profile suitable for therapeutic use and do not exert toxicity toward 16HBE cells or HDFs at their respective MIC_90_ values. The safety, stability, and potent antimicrobial activity of C58 and its derivatives make these compounds promising candidates for antimicrobial therapy. Here, we have demonstrated that these compounds are non-toxic at therapeutic concentrations; however, therapeutics must be non-toxic both *in vitro* and *in vivo*. These *in vitro* findings underscore the need for further characterization studies that will delineate potential toxicity. We will confirm the safety profile of these compounds *in vivo* before further activity studies are conducted.

### Mechanism of action

We have assessed the *in vitro* antimicrobial activity and safety profile of these compounds. To fully characterize a potential antimicrobial agent, it is necessary to investigate its mechanism of action. Our results suggest that there is a low probability of resistance development; therefore, it is possible that the compounds bind to a highly conserved region of a protein that is highly conserved across Gram-positive species. The morphological changes observed in the TEM images suggest that a cell division process may be a target of these compounds. We hypothesize that filamentous temperature-sensitive protein Z (FtsZ), which plays an essential role in *S. aureus* cell division and septal proteoglycan synthesis, is inhibited or dysregulated in the presence of C59 (26). FtsZ is a bacterial homolog of tubulin and has been widely recognized as the basic structural component of the cytokinetic ring structure required for *S. aureus* cell division (27). FtsZ acts as a scaffold for recruiting the proteins necessary to form the cell division complex, also known as the divisome (28–30). The FtsZ scaffold is formed via polymerization at the midcell, resulting in a dynamic ring structure at the site of division, commonly referred to as the Z-ring (31–33).

Previous studies have reported that the treatment of MRSA with an FtsZ inhibitor (PC190723) resulted in FtsZ delocalization, which led to aberrant septum formation either above or below the midcell or at the cell periphery (34). Another report demonstrated that the treatment of MRSA with a FtsZ inhibitor (TXA707) resulted in FtsZ localization away from the midcell and dysfunctional septation, which gave rise to incomplete or aberrant septal structures (35). Additionally, these reports have demonstrated that treatment with TXA707 leads to the disruption of normal septum formation, resulting in oblong or abnormally shaped cells (35). Another study has also reported that the treatment of MRSA with an FtsZ inhibitor (compound 28) resulted in Z-ring delocalization from the midcell (36, 37) . Our observations of incomplete or aberrant septation and abnormal cell shapes suggest that C59 treatment may contribute to FtsZ inhibition or dysregulation; however, additional investigation is warranted to determine the precise targets and mechanisms of action (18). Future studies will elucidate the mechanism of action of these compounds and determine if FtsZ is the target of this novel class of antimicrobials. Once we have elucidated the mechanism of action of C58 and its derivatives, we may make rational modifications to improve binding.

### Future studies and conclusion

In summary, we report the total synthesis of the natural compound C58 and its structural analog, C59, as well as the successful modification of C59 to produce its water-soluble sodium salt (C59Na). We have demonstrated that these compounds exhibit potent antimicrobial activity against MRSA in both planktonic and biofilm growth modes. Additionally, we observed that C59 demonstrates a low propensity for resistance acquisition. At therapeutic concentrations, C58 and its derivatives do not exert toxicity against the two mammalian cell lines tested, nor do they exhibit hemolytic activity against erythrocytes. Finally, to elucidate the mechanism of action, further investigation of the interactions between C58 and its derivatives with MRSA will be conducted to determine if the compounds bind to FtsZ and interfere with cell division.

The *in vitro* antimicrobial activity of the compounds was assessed under conditions that we believe mimic the milieu at the site of infection, through the alteration of pH, incubation under constant shaking and aeration, and incubation with persistently sub-inhibitory concentrations. However, the *in vivo* environment is dynamic and complex, and the *in vitro* studies we have presented do not fully recapitulate the myriad of variables that can influence the safety and activity of an antibiotic agent. Our results provide a foundation for the future investigation of these compounds, which will include a comprehensive evaluation of their activity *in vivo*. We will determine their pharmacokinetic and pharmacodynamic properties and study their biodistribution, which will offer invaluable insights into their therapeutic potential. Water-soluble formulations such as C59Na are often preferred for clinical use, as they can be easily administered and have enhanced bioavailability. Additionally, to optimize the hydrophobic C58 and C59 for use clinically, future studies will involve the encapsulation of these compounds within a drug delivery system, such as a polymeric nanoparticle formulation, which will augment delivery and the potential of these compounds as effective antimicrobial agents.

In conclusion, we present the total synthesis of the natural product C58 and its derivatives that constitute a novel class of antibiotics, which exhibit potent antimicrobial activity against MRSA and other Gram-positive species. In our future investigations, we will confirm the *in vitro* antimicrobial activity and safety we have observed by recapitulating these studies *in vivo*. These compounds hold tremendous potential as next-generation antimicrobials for the treatment of infections with resistant Gram-positive pathogens and offer a potential solution to combat the escalating threat of antibiotic resistance.

## MATERIALS AND METHODS

### Materials

Bromine, thionyl chloride, 2,2′-biphenol, sodium hydride, calcium dichloride, iodine, *n*-butyl lithium, potassium hydride, triethyl amine, acetic anhydride, dichloromethane, toluene, dimethylsulfoxide (DMSO), tetrahydrofuran (THF), methanol, vancomycin, daptomycin, and linezolid were purchased from Sigma Aldrich. Clindamycin and trimethoprim-sulfamethoxazole were purchased from Gold Biotechnology. Minimum essential medium (MEM) with Earle’s balanced salts, Dulbecco’s modified Eagle’s medium (DMEM), penicillin–streptomycin, and L-glutamine were also purchased from Sigma Aldrich. Fetal bovine serum was purchased from GenClone. Tryptic soy agar (TSA) plates, Tryptic soy broth (TSB), Mueller Hinton (MH) broth, Luria–Bertani (LB) broth, and blood agar plates were procured from Becton Dickinson. Sheep blood was purchased from Remel. The alamarBlue Cell Viability Assay was purchased from Thermo Scientific. Clinical isolates of MRSA were collected at Washington University in St. Louis and are available by request. All *Staphylococcus epidermidis* isolates were acquired from BEI Resources. 16HBEs (16HBE14o-) were generously provided by Dr. D. Gruenert (University of California, San Francisco, CA) and are a human bronchial epithelial cell line transformed with SV40 large T-antigen using the replication-defective pSVori plasmid (38). Human dermal fibroblasts at passage 4 were generously donated by Dr. C. Tamminga (University of Texas Southwestern Medical Center, Dallas, TX).

### Synthesis of c58 and c59

**Fig 7:**
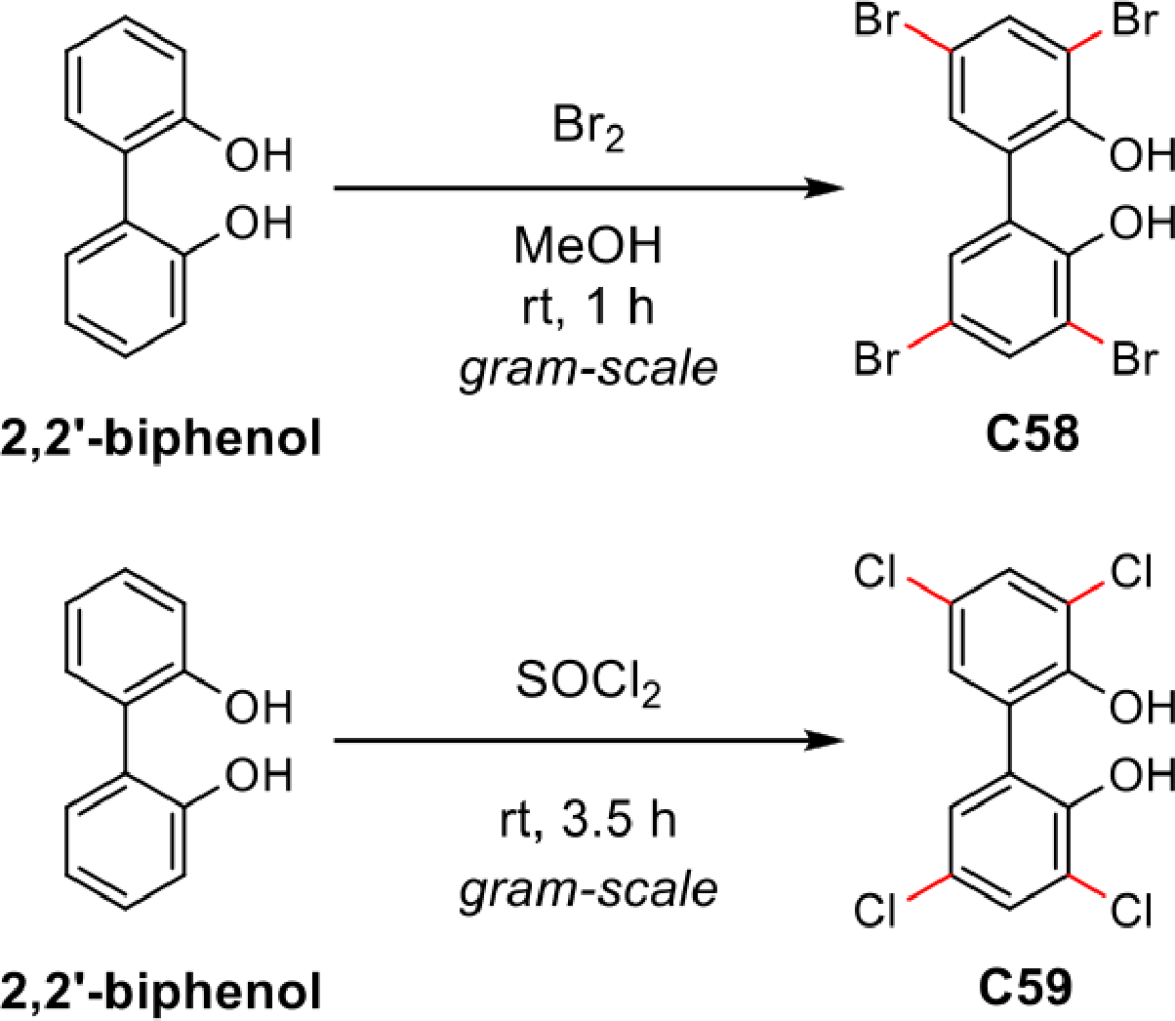
Gram-scale syntheses of C58 (MC21-A) and C59.

Syntheses for tetra-halogenated 2,2′-dihydroxybiphenyl compounds similar to C58 and C59 have been reported as far back as the early 1900s (39). More recently, efficient syntheses that utilize either Br_2_ or SOCl_2_ as halogenating agents to furnish these types of structures for asymmetric catalysis applications have been reported (16). These modern methods can achieve complete selective halogenation (i.e., halogenation *ortho* and *para* to the phenolic position) (Fig. 7). The desired compounds, C58 and C59, were synthesized according to the reported procedures (16). The ^1^H and ^13^C NMR of these compounds matched those reported in the literature (16).

### Synthesis of salts of C59

**Fig 8:**
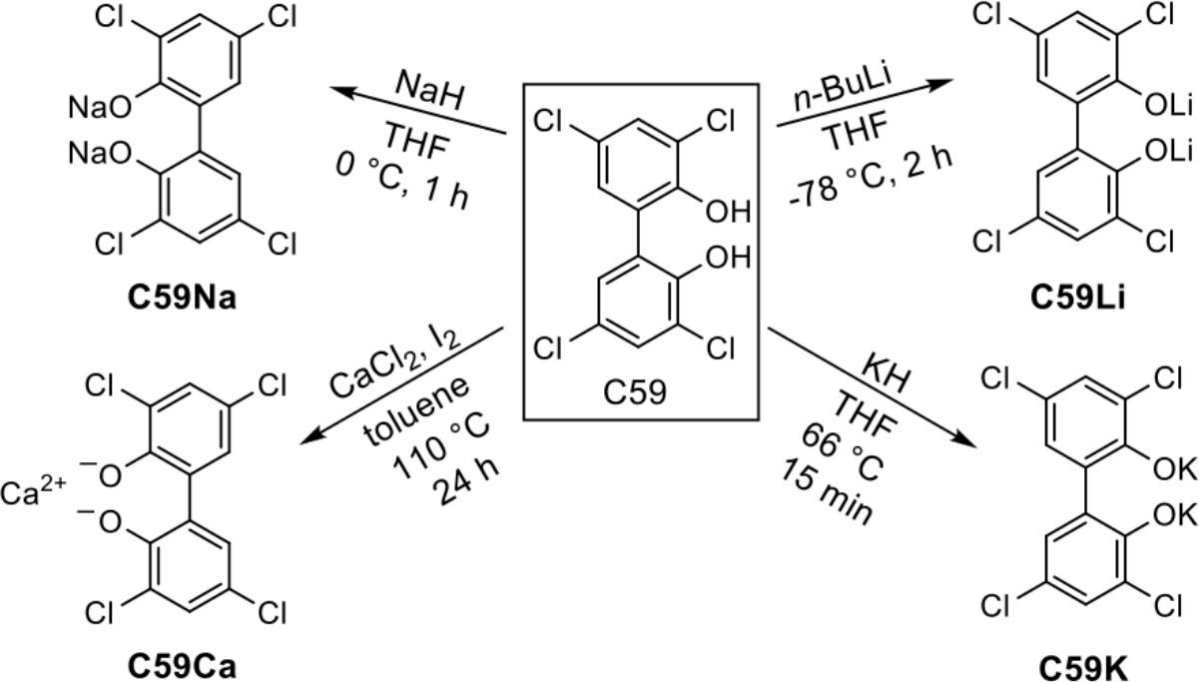
Synthesis of the salts of C59.

To compare the activities of various C59 salts, adapted literature procedures were used to synthesize the sodium, lithium, calcium, and potassium salts of C59 (Fig. 8). The modified literature procedures produced the desired salts in moderate to good yields (21%–89%) (40–42).

### Synthesis of the sodium salt of C59

For the sodium salt of C59 (C59Na), 60% NaH dispersed in mineral oil (48 mg, 1.2 mmol, 2.4 equiv.) was suspended in THF (1 mL) and cooled to 0°C. 2,2′-Dihydroxybiphenyl (162 mg, 0.5 mmol, 1.0 equiv.) dissolved in THF (1 mL) was slowly added to the solution at 0°C and left stirring. After 1 hour, the reaction was removed from the ice bath, filtered through celite, and washed with Et_2_O. The filtrate was concentrated, and the resulting solid was washed with pentane (40). The solid was dried with a vacuum to produce 165 mg of the desired C59Na salt as a yellow solid (89% yield). ^1^H NMR (600 MHz, acetone-*d*_6_) δ 6.95 (s, 2H), 6.63 (s, 2H).

### Synthesis of the calcium salt of C59

For C59Ca, 2,2′-dihydroxybiphenyl (162 mg, 0.5 mmol, 1.0 equiv.), CaCl_2_ (66 mg, 0.6 mmol, 1.2 equiv.), and I_2_ (4 mg, 0.016 mmol, 3.3 mol%) were dissolved in toluene (2.5 mL, 0.2 M). The mixture was heated to reflux and left stirring for 24 hours. The reaction was then removed from heat, allowed to cool to room temperature, and filtered (41). The resulting solid was dried under vacuum to produce 39.4 mg of the desired C59Ca salt as a brown solid (21% yield). ^1^H NMR (600 MHz, acetone-*d*_6_) δ 7.28 (d, *J* = 2.6 Hz, 2H), 7.17 (d, *J* = 2.7 Hz, 2H).

### Synthesis of the lithium salt of C59

For C59Li, 2,2′-dihydroxybiphenyl (162 mg, 0.5 mmol, 1.0 equiv.) was dissolved in THF (2.5 mL, 0.2 M) and cooled to −78°C. *n*-BuLi (1.6 M in hexanes, 656 L, 1.05 mmol, 2.1 equiv.) was added dropwise to the reaction mixture. After stirring for 2 hours, the reaction was removed from the cold bath and allowed to warm to room temperature. The reaction solution was concentrated, and the resulting solid was washed with pentane (42). The solid was dried under vacuum to produce 102 mg of the desired C59Li salt as a yellow solid (61% yield). ^1^H NMR (600 MHz, acetone-*d*_6_) δ 7.03 (s, 2H), 6.73 (s, 2H).

### Synthesis of the potassium salt of C59

For C59K, 2,2′-dihydroxybiphenyl (162 mg, 0.5 mmol, 1.0 equiv.) and KH (122 mg, 3.05 mmol, 6.0 equiv.) were dissolved in THF (2.5 mL, 0.2 M). The mixture was heated to reflux and left stirring for 24 hours. The reaction was then removed from heat, allowed to cool to room temperature, and filtered (43). The resulting solid was dried under vacuum to produce 154 mg of the desired C59K salt as a yellow solid (77% yield). ^1^H NMR (600 MHz, acetone-*d*_6_) δ 6.98 (d, *J* = 3.0 Hz, 2H), 6.73 (dd, *J* = 3.0, 1.6 Hz, 2H).

### Synthesis of the acetate of C59

For C59Ac, 3,3′,5,5′-tetrachloro-[1,1′-biphenyl]−2,2′-diol (3.2 g, 10 mmol, 1.0 equiv.) and triethylamine (4.05 g, 40 mmol, 4.0 equiv.) were dissolved in dichloromethane. Acetic anhydride (4.08 g, 40 mmol, 4.0 equiv.) was added dropwise to the stirring solution at room temperature. After stirring for 9 hours, the reaction solution was washed with 1 M HCl, sodium carbonate, and water. The organic layer was dried over MgSO_4_ and concentrated. The resulting colorless oil was purified via Biotage flash column chromatography (3% EtOAc/Hex to 15% EtOAc/Hex) to provide 3.56 g of C59Ac as a white sticky solid (87% yield) (44). ^1^H NMR (nuclear magnetic resonance) (600 MHz, CDCl_3_) δ 7.51 (d, *J* = 2.4 Hz, 2H), 7.17 (d, *J* = 2.4 Hz, 2H), 2.14 (s, 6H); ^13^C NMR (151 MHz, CDCl_3_) δ 167.74, 143.71, 132.17, 131.91, 130.55, 129.38, 129.11, 20.36.

### Characterization of C58, C59, and the salts of C59

The pH values for the aqueous solutions of C58, C59, the salts of C59 (i.e., C59Na, C59Ca, C59Li, and C59K), and C59Ac were determined using a Mettler Toledo FE20/EL20 pH meter. The compounds were placed in borosilicate glass vials and reconstituted in 10 mg/mL of distilled deionized water. The resulting solutions ranged from sparingly soluble to partially soluble. The final pH values represent the average of three values measured with the pH probe at room temperature (22.9°C).

### Determination of minimum inhibitory and bactericidal concentrations

MIC and MBC values for all compounds were determined against clinical and laboratory isolates of MRSA and *Staphylococcus epidermidis* using the standard CLSI broth microdilution method. Briefly, frozen bacterial stocks of MRSA or *S. epidermidis* were streaked onto TSA plates or blood agar plates, respectively, and incubated overnight at 37°C. The bacterial suspension was further diluted using MH broth to a final concentration of 5E5 CFU/mL. Subsequently, a single colony was selected and cultured in MH broth to Optical Density (OD)_650_ = 0.4. A 10-mg/mL solution of C58, C59, or linezolid was prepared in DMSO, and serial dilutions were performed in MH broth. Similarly, 10 mg/mL vancomycin, daptomycin, and clindamycin stocks were prepared in distilled, deionized water, and serial dilutions were performed in MH broth (or MH broth supplemented with 50 mg/L of Ca^2+^ for daptomycin). A 100-µL drug solution was then mixed with 100-µL bacterial suspension to yield a final drug concentration between 0.06 and 32 µg/mL with 2.5% DMSO. Bacteria were incubated for 18–20 hours, and the lowest drug concentration that inhibited bacterial growth was designated as the MIC. The drug and bacterial solutions were then plated onto blood agar plates and incubated at 37°C for an additional 24 hours. Finally, the plates were visually inspected for bacterial growth, and the lowest drug concentration without any bacterial growth was designated as the MBC. All experiments were performed in triplicate, and the highest MIC and MBC values were reported. The MIC_90_ and MBC_90_ values were calculated as the concentration of drug required to inhibit and eradicate 90% of the tested bacterial isolates. MIC and MBC experiments were also repeated with MH broth adjusted to pH 5.5, 6.0, 6.5, and 7.0.

### Kill kinetics against stationary-phase bacteria

MRSA isolates, TCH 1516 and SA LL 06, were grown to the stationary phase, and the rate of bacterial killing was determined. Frozen bacterial stocks were streaked onto TSA plates and grown overnight. A single colony was selected and incubated overnight in LB broth at 37°C with constant orbital shaking at 200 RPM. Next, bacterial suspension was washed twice with 150 mM sodium chloride and resuspended in MH broth at a concentration of 1.2E9 CFU/mL. Next, solutions that had a concentration of 1×, 2×, 4×, 8×, or 16× MIC of C59 or vancomycin were mixed with 2E6 CFU/mL bacteria. Glass tubes containing 5 mL of bacteria and drug solutions were grown in a shaking incubator at 37°C. At 0, 4, 8, and 24 hours, bacterial suspensions were collected, serially diluted, and plated onto a blood agar plate. Bacteria were allowed to grow for 24 hours and were enumerated to yield colony-forming units per milliliter.

### Antimicrobial activity against biofilm mode bacteria

The antimicrobial activities of C58, C59, and vancomycin were evaluated using an MBEC assay according to the manufacturer’s recommended protocol (lnnovotech, Edmonton, AB, Canada). The frozen bacterial stock was streaked onto TSA plates and grown as described above. After 18–24 hours, bacteria were resuspended in TSB to OD_650_ = 0.4 and diluted to 1E7 CFU/mL, and 150 µL of the bacterial suspension was added to each well in an MBEC plate. The plates were incubated at 37°C on a rocker for 72 hours to allow biofilms to form on the pegs. Established biofilms were washed twice with 150 mM saline to remove any planktonic-phase bacteria and were incubated with the drug solution for up to 24 hours. Biofilms were washed again with saline and recovered in TSB by sonication. Finally, the bacterial solution was serially diluted and plated onto blood agar plates. Plates were incubated at 37°C for 24 hours, and bacterial colonies were counted to determine the number of colony-forming units per milliliter. These results were confirmed using crystal violet staining. Bacterial biofilms were established, treated with antimicrobial agents, washed, and harvested by sonication, as described above. The bacteria were then stained using crystal violet, and absorbance was measured at 450 nm using a BioTek Cytation 5 multi-mode reader.

### Determination of risk for resistance development

The probability of resistance development against antimicrobial agents was determined using a single-step method and a multi-step serial dilution study.

#### Single-step resistance development

For the single-step study, bacteria were grown to the stationary phase as described above. Bacteria at a concentration of 1E9 CFU were then plated onto C59 and vancomycin-loaded plates containing 1×, 2×, 4×, and 8× MIC concentrations of the therapeutic agents with 1% DMSO. Bacteria were incubated at 37°C for 48 hours, and colony counts were enumerated to calculate the frequency of resistance development, as described in the literature (45, 46).

#### Resistance acquisition during serial passage study

MRSA isolate TCH 1516 was grown to the stationary phase (OD_650_ = 1.0) in 10 mL of MH broth. A 1:100 dilution was performed with fresh MH broth containing 0–256× MIC of the therapeutic with 2.5% DMSO and incubated at 37°C with orbital shaking at 200 RPM for 24 hours. The second highest drug concentration resulting in bacterial growth (OD_650_ >1.0) was used as the inoculum after a 1:1,000 dilution for subsequent steps, every 24 hours, for up to 21 days.

### Hemolytic activity of compounds

A 100-µL volume of 1% sheep blood prepared in 0.9% saline and containing 0, 2, 4, 8, 16, 24, 32, 48, and 64 µg/mL of C58, C59, or C59Na with 2.5% DMSO was incubated in a 96-well plate for 1 hour at 37°C with continuous orbital shaking at 120 RPM. Erythrocytes were also incubated with drugs with known hemolytic activity, amphotericin B and gramicidin D, at similar drug concentrations. The positive control consisted of red blood cells in sheep blood completely lysed with 0.1% Triton-X solution. Next, the 96-well plate was centrifuged to separate the red blood cells, the supernatant was transferred to a fresh 96-well plate, and absorbance was measured at 405 nm using a BioTek Cytation 5 multi-mode reader. The percentage of hemolyzed red blood cells or erythrocyte hemolysis was calculated using the following formula: % hemolysis = (*A*_S_ – *A*_B_)/(*A*_PC_ – *A*_PBC_), where *A*_S_ is the absorbance of the sample, *A*_B_ is the blank absorbance or negative control absorbance, *A*_PC_ is the absorbance of positive control (100% hemolysis), and *A*_PBC_ is the absorbance of positive control blank solution.

### Toxicity of compounds against human bronchial epithelial and fibroblast cells

16HBEs and HDFs were selected for toxicity studies because MRSA commonly infects the lungs and skin, respectively. 16HBEs were cultured with MEM with Earle’s balanced salts and non-essential amino acids supplemented with penicillin–streptomycin (1%), L-glutamine (1%), and fetal bovine serum (10%). HDFs were cultured using DMEM supplemented with penicillin–streptomycin (1%), L-glutamine (1%), and fetal bovine serum (10%). 16HBEs were used between passages 20 and 30, while HDFs were used between passages 7 and 12. 16HBEs and fibroblasts were seeded at a density of 25,000 and 15,000 cells/well, respectively, with 100 µL feeding media, in a 96-well plate. Cells were incubated for 24 hours at 37°C with 95% relative humidity and 5% carbon dioxide. Next, the media were aspirated, and 100-µL media containing 0–200-µM drug with 2.5% DMSO were added to each well. Cells were incubated with the drug solution for 24 hours, and an alamarBlue cell viability assay was then performed according to the manufacturer’s protocol.

### Transmission electron microscopy

Bacterial samples were prepared and imaged according to a previously published protocol (47). Briefly, MRSA isolate TCH 1516 was treated with 0, 0.5, and 1.0 µg/mL C59 and was pelleted and fixed in 2.5% glutaraldehyde and 1% acrolein in 0.2 M Sorensen’s phosphate buffer for 1 hour at room temperature. The fixed samples were stained with 1% aqueous osmium tetroxide overnight at 4°C, dehydrated with acetone, and then infiltrated with resin. Samples were thin-sectioned with a microtome (Boeckeler MTX), poststained with uranyl acetate and lead citrate, and imaged using a JEOL 1200 EX electron microscope.

### Statistical analysis

All statistical analyses were conducted using Prism 10 GraphPad Software, Inc (San Diego, CA). All data from each experiment have been obtained from biological replicates to ensure reproducibility. Data are represented as the mean with standard error of the mean. MIC and MBC data are repeated measurements from three biological replicates. Kill kinetics, cellular toxicity, and hemolysis data have been analyzed using two-way ANOVA with multiple comparisons (^****^P < 0.0001, ^***^P < 0.001, ^**^P < 0.01, and ^*^P < 0.05).

## SUPPLEMENTAL MATERIAL

Supplementary figures and tables spectrum.01515-22-s0001.pdf Fig. S1 to S4 and Tables S1 to S5.

ASM does not own the copyrights to Supplemental Material that may be linked to, or accessed through, an article. The authors have granted ASM a non-exclusive, world-wide license to publish the Supplemental Material files. Please contact the corresponding author directly for reuse.
