# Supplementary material for: Antimicrobial activity of a natural compound and analogs against multi-drug-resistant Gram-positive pathogens
Source: Microbiol Spectr. 2024 Jan 30;12(3):e01515-22. doi: 10.1128/spectrum.01515-22 (PMC10913730; doi:10.1128/spectrum.01515-22)
Supplement: Supplementary figures and tables — Fig. S1 to S4 and Tables S1 to S5. [file spectrum.01515-22-s0001.pdf]

Supplementary Information for

Antimicrobial Activity of a Natural Compound and Analogs Against Multi-Drug Resistant Gram-positive Pathogens

Kush N. Shah, PhD,[a]# Parth N. Shah, PhD,[a]# Francesca O. Agobe, BS, [a]# Kaitlyn Lovato, PhD,[c] Hongyin Gao, PhD,[c] Oluwadara Ogun, MBIOT,[a] Cason Hoffman, BS,[a] Marium Yabe-Gill, MD,[b] Qingquan Chen, PhD,[a] Jordan Sweatt, BS,[a] Bhagath Chirra, PhD,[a] Ricardo Munoz-Medina, MS, [a] Delaney E. Farmer, BS, [a] László Kürti, PhD,[c]# Carolyn L. Cannon, MD, PhD[a]#

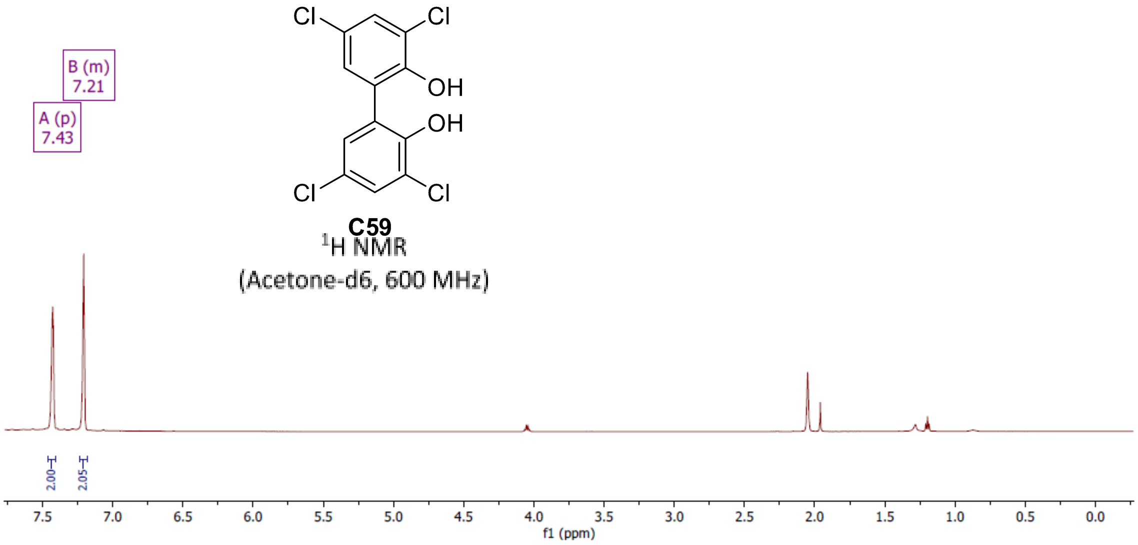

B (m)
7.21
A (p)
7.43
C59
^{1}H NMR
(Acetone-d6, 600 MHz)
2.00
2.05
7.5 7.0 6.5 6.0 5.5 5.0 4.5 4.0 3.5 3.0 2.5 2.0 1.5 1.0 0.5 0.0
f1 (ppm)

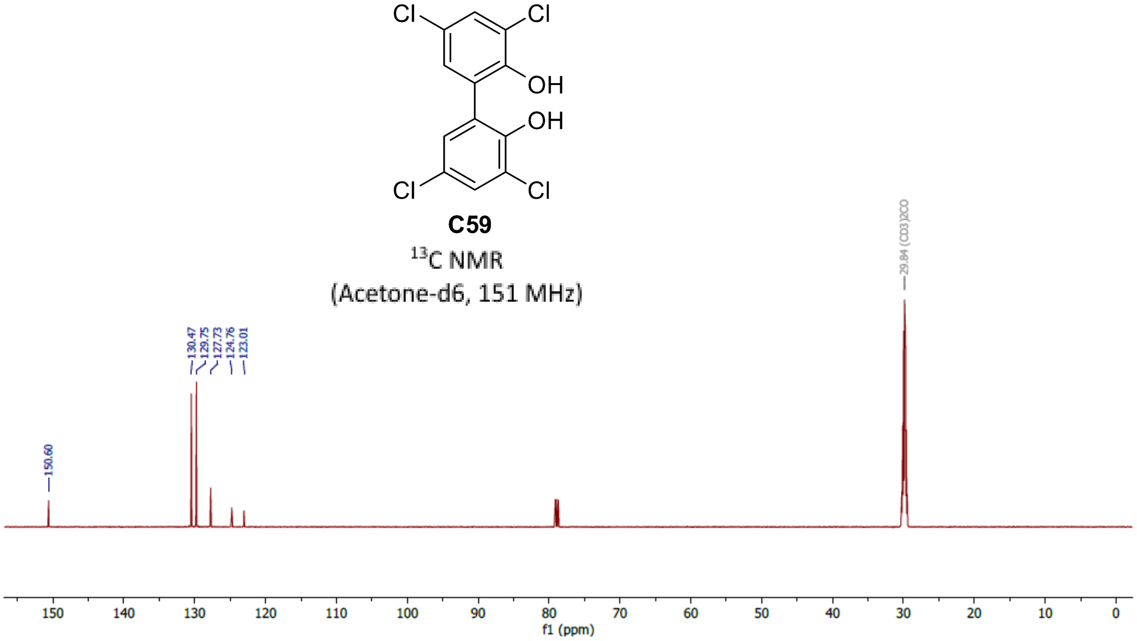

C59
^{13}C NMR
(Acetone-d6, 151 MHz)
150.60
130.47
129.75
127.73
124.76
123.01
29.84 (CD3)2CO
150 140 130 120 110 100 90 80 70 60 50 40 30 20 10 0
f1 (ppm)

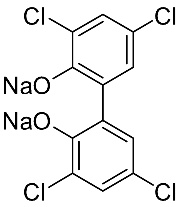

C59Na

^{1}H NMR

(Acetone-d6, 600 MHz)

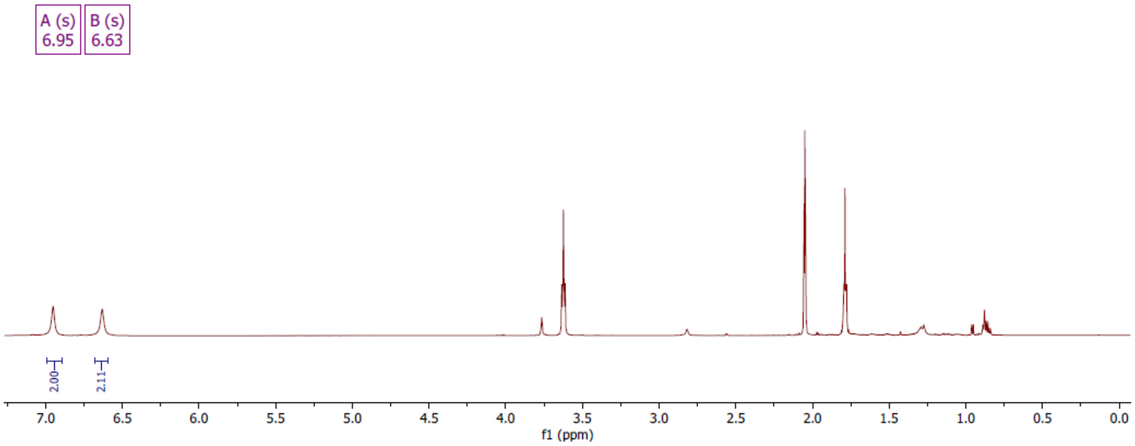

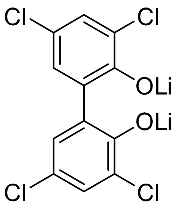

C59Li

^{1}H NMR

(Acetone-d6, 600 MHz)

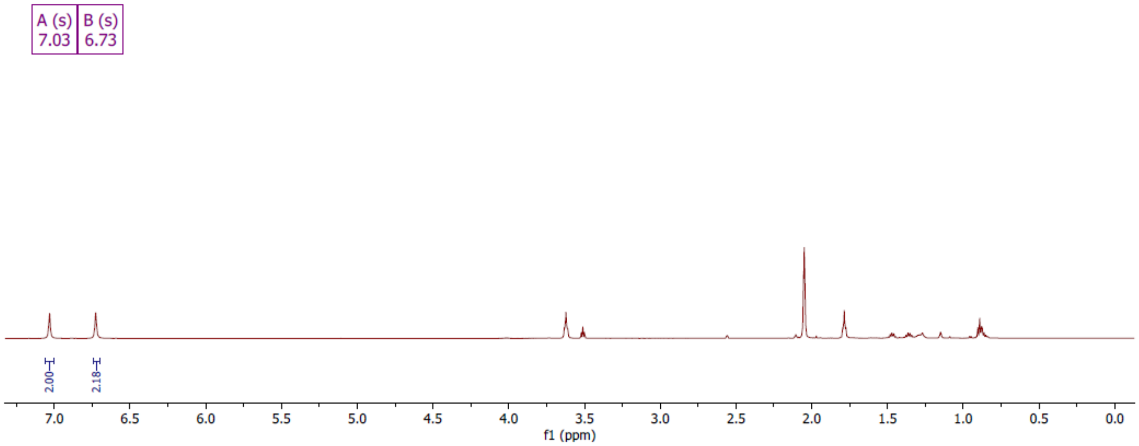

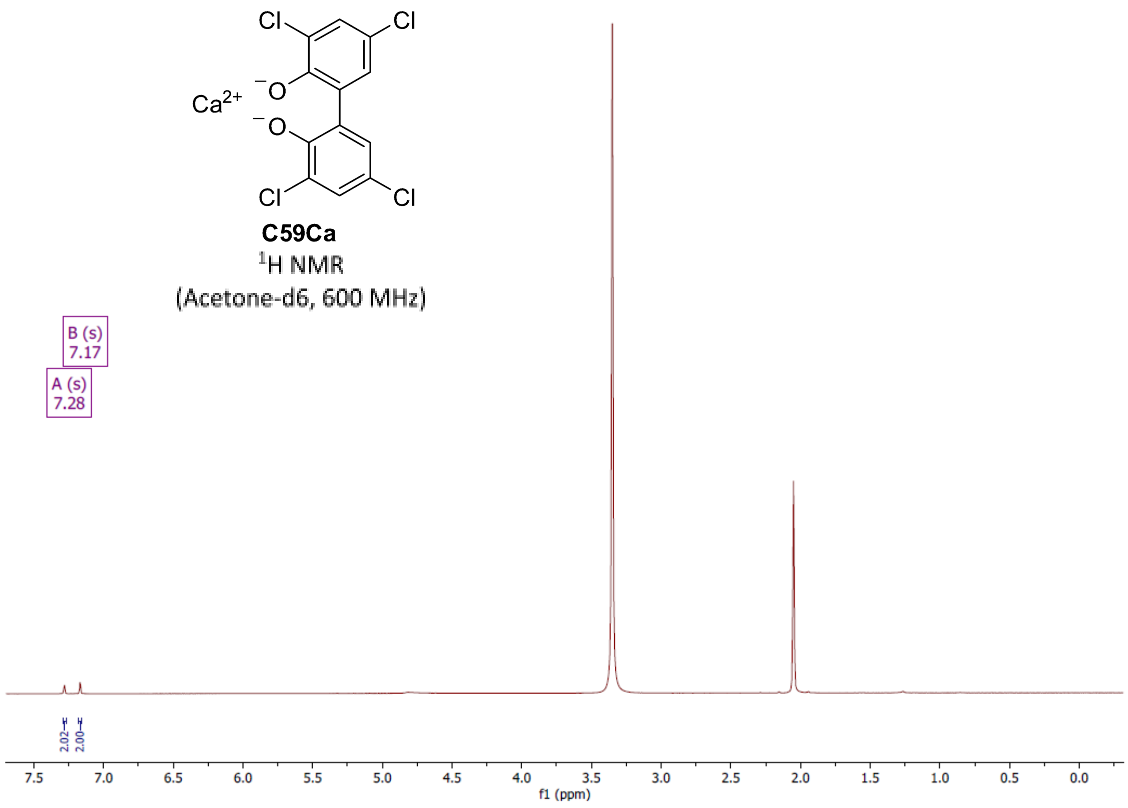

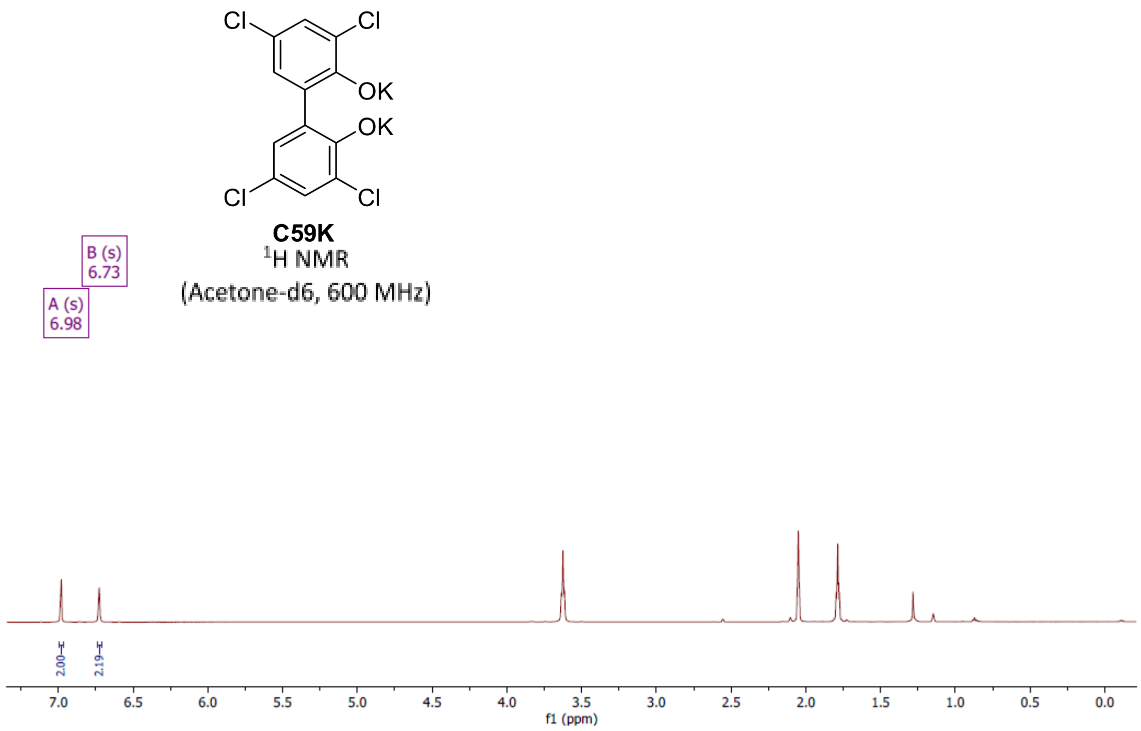

Figure S1. ^{1}H and ^{13}C NMR spectra of the compounds C59, C59Na, C59Li, C59Ca, and C59K.

Table S1. Minimum concentration resulting in inhibition or eradication of the 6 selected MRSA isolates at pH 7.4. All concentrations are in µg/mL.

Isolate	TCH 1516		MRSA 0608		MRSA 0646		MRSA 0641		MRSA 0606		MRSA 0634	
**Drug**	MIC	MBC	MIC	MBC	MIC	MBC	MIC	MBC	MIC	MBC	MIC	MBC
C59	2	4	2	6	2	4	2	8	2	4	2	8
C59Na	2	2	2	4	1	4	2	4	2	2	2	2
C59K	2	4	4	4	2	4	2	4	2	6	2	6
C59Li	2	2	2	6	1	4	2	4	1	2	2	2
C59Ca	2	6	2	6	2	6	2	4	2	4	2	6
C59Ac	4	4	4	12	4	8	4	12	4	8	4	4

Table S2. Minimum concentration resulting in inhibition or eradication of 90% of the 21 evaluated *Staphylococcus epidermidis* isolates (MIC_{90} and MBC_{90}) determined using CLSI broth microdilution technique. Strains BCM0060, E13A, M0881, M23864:W2, NIH04008, NIH05001, NIHLM001, NIHLM003, NIHLM008, NIHLM015, NIHLM018, NIHLM020, NIHLM031, NIHLM087, NRS7, NRS8 (HIP4680), NRS34, NRS122, NRS858 (VCU071), SK135, and W23144.

Drug	MIC_{90} (µg/mL)	MBC_{90} (µg/mL)
C58	2 (range 1-4)	8 (range 4-12)
C59	4 (range 2-4)	12 (range 6-12)
Vancomycin	4 (range 1-8)	8 (range 2-16)

Table S3. Minimum inhibitory and bactericidal concentrations (MIC and MBC) of C58 and C59 against other Gram-positive species and Gram-negative species. ND = not done

Drug	C58		C59	
	MIC (µg/mL)	MBC (µg/mL)	MIC (µg/mL)	MBC (µg/mL)
Enterococcus faecalis (ATCC 49532)	6	12	8	16
Enterococcus faecalis (ATCC 51575)	6	12	8	16
Streptococcus pyogenes (NR1571)	4	16	6	24
Streptococcus pyogenes (NR1575)	4	12	6	16
Pseudomonas aeruginosa (PAO1)	>32	ND	>32	ND
Pseudomonas aeruginosa (PAM57-15)	>32	ND	>32	ND

Table S4. Minimum inhibitory concentration (MIC, μg/mL) of test antimicrobials, C58, C59, C59Na, C59Li, compared with vancomycin against six MRSA isolates at pH 5.5, 6.5, and 7.4.

Drug	pH	TCH 1516	MRSA 0608	MRSA 0646	MRSA 0641	MRSA 0606	MRSA 0634	Average
C58	5.5	1	0.5	0.5	0.5	0.5	0.5	0.58
	6.5	1	2	1	1	1	1	1.2
	7.4	2	2	2	2	2	2	2.0
C59	5.5	0.5	0.5	0.5	0.5	0.5	0.5	0.50
	6.5	1	2	1	1	1	1	1.2
	7.4	2	2	2	2	2	2	2.0
C59Na	5.5	0.25	0.25	0.25	0.25	0.25	0.25	0.25
	6.5	1	1	0.5	0.5	0.5	1	0.75
	7.4	2	2	1	2	2	2	1.8
C59Li	5.5	0.25	0.25	0.25	0.25	0.25	0.25	0.25
	6.5	1	1	0.5	0.5	1	1	0.83
	7.4	2	2	1	2	1	2	1.7
Vancomycin	5.5	2	4	2	2	2	2	2.3
	6.5	1	2	2	1	1	2	1.5
	7.4	1	2	2	1	2	2	1.7

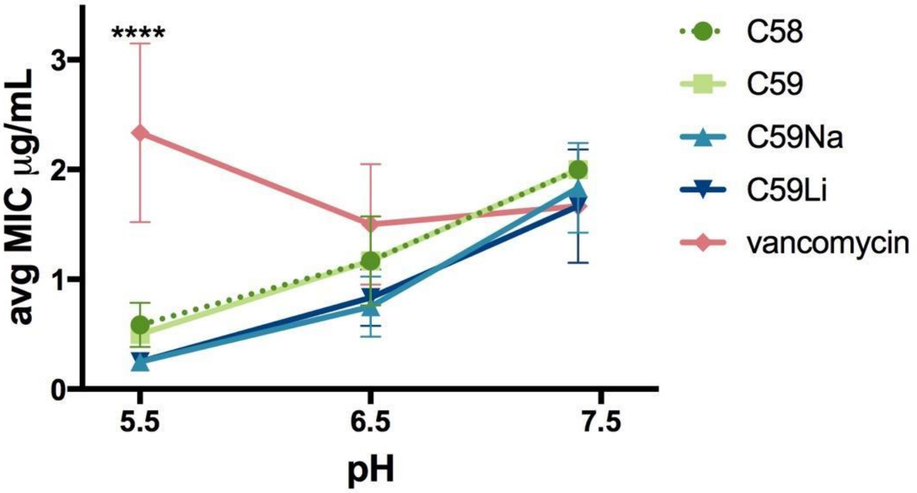

Figure S2. Average minimum inhibitory concentration (avg MIC, μg/mL) of test antimicrobials, C58, C59, C59Na, C59Li, compared with vancomycin against six MRSA isolates at pH 5.5, 6.5, and 7.4. Data shown as mean +/- SD. Vancomycin versus each of C58, C59, C59Na, and C59Li by two-way ANOVA with Sidak's multiple comparison test (n = 6 isolates). **** $p < 0.0001$

Table S5. Minimum bactericidal concentration (MBC, μg/mL) of test antimicrobials, C58, C59, C59Na, C59Li, compared with vancomycin against six MRSA isolates at pH 5.5, 6.5, and 7.4.

Drug	pH	TCH 1516	MRSA 0608	MRSA 0646	MRSA 0641	MRSA 0606	MRSA 0634	Average
**C58**	5.5	1	0.5	1	0.5	0.5	0.5	0.67
	6.5	2	2	2	2	2	2	2.0
	7.4	4	4	4	4	6	2	4.0
**C59**	5.5	0.5	0.5	0.5	0.5	0.5	0.5	0.50
	6.5	4	2	4	4	6	4	4.0
	7.4	4	6	4	8	4	8	5.7
**C59Na**	5.5	0.25	0.25	0.25	0.25	0.25	0.25	0.25
	6.5	2	1	1	1	1	1	1.2
	7.4	2	4	4	4	2	2	3.0
**C59Li**	5.5	0.25	0.25	0.25	0.25	0.25	0.25	0.25
	6.5	1	2	1	6	1	1	2.0
	7.4	2	6	4	4	2	2	3.3
**Vancomycin**	5.5	4	4	8	2	2	8	4.7
	6.5	2	2	2	2	4	4	2.3
	7.4	1	6	6	4	2	6	4.2

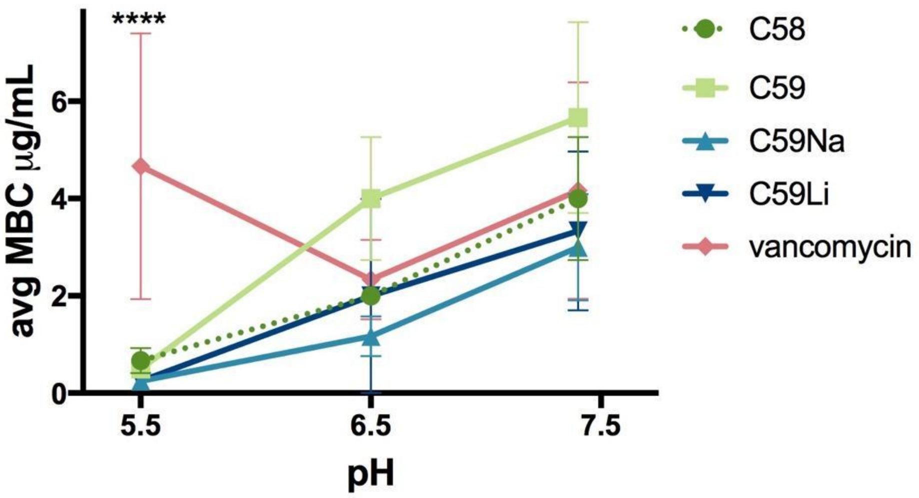

Figure S3. Average minimum bactericidal concentration (avg MBC, μg/mL) of test antimicrobials, C58, C59, C59Na, C59Li, compared with vancomycin against six MRSA isolates at pH 5.5, 6.5, and 7.4. Data shown as mean +/- SD. Vancomycin versus each of C58, C59, C59Na, and C59Li by two-way ANOVA with Sidak's multiple comparison test (n = 6 isolates). **** $p < 0.0001$

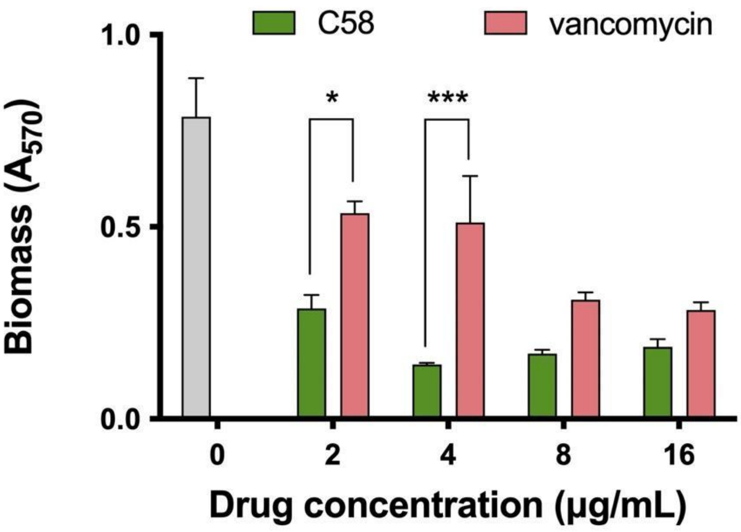

Figure S4. Absorbance measurements from crystal violet-stained MRSA biofilms (SAD05) upon incubation for 72 hours with either C58 or vancomycin. Data shown as mean +/- SD. Vancomycin versus C58 by two-way ANOVA with Sidak's multiple comparison test (n = 5 replicates). * $p = 0.029$, *** $p < 0.0005$
